# Normative Values of the Groffman Visual Tracing Test for the Assessment of Oculomotor Performance in the Adult Population

**DOI:** 10.3390/vision6020034

**Published:** 2022-06-15

**Authors:** Alessio Facchin, Elisa Mischi, Camilla Iannello, Silvio Maffioletti, Roberta Daini

**Affiliations:** 1 Department of Psychology, University of Milano-Bicocca, 20126 Milan, Italy; roberta.daini@unimib.it; 2 COMiB—Optics and Optometry Research Center, University of Milano-Bicocca, 20126 Milan, Italy; 3 NeuroMI—Milan Center for Neuroscience, 20126 Milan, Italy; 4 Institute of Research and Studies in Optics and Optometry, 50059 Vinci, Italy; silvio.maffioletti@gmail.com; 5 Material Science Department, University of Milano-Bicocca, 20126 Milan, Italy; e.mischi@campus.unimib.it (E.M.); c.iannello2@campus.unimib.it (C.I.)

**Keywords:** eye movements, oculomotor performance, oculomotor dysfunction, learning disabilities, aging, stroke, visual tracing

## Abstract

The Groffman visual tracing (GVT) test is an indirect psychometric measure of oculomotor performance, used for the clinical assessment of eye movements. The test consists of two cards with five contorted lines of increasing overlap, crowding, and difficulty. The task starts from each of the letters at the top of the page, follows the line from the letter to the corresponding number at the bottom of the page, and the number is named. Although the GVT test was developed for the evaluation of children, it has also been applied to adults with visual and cognitive deficits. However, it lacks reference values. Therefore, the aim of the study was to assess oculomotor behavior across the typical human lifespan and to define normative data in an adult population. A total of 526 adults aged between 20 and 79 years, all without neurological or psychiatric deficits, were enrolled in the study. The results were analyzed by considering the accuracy and execution times separately. An influence of age, education and sex for accuracy was found, and age for the execution times was found. Norms for adults were developed considering the specific structure of the test and the accuracy and the execution time separately. The GVT test can now be applied in healthy and neurological adult populations for the evaluation of oculomotor performance.

## 1. Introduction

The act of following a line with the eyes, although a seemingly simple task, requires several skills and the application of different cognitive functions. This task was formerly called visual tracing [1], in contrast to visual tracking, which refers to the detection of a target in motion [2].

A simple clinical task that uses visual tracing is the Groffman visual tracing (GVT) test. Initially developed by Groffman in 1966 [3] to assess the tracing abilities of children, it has recently become the subject of some research into its use as a clinical tool or experimental task in both children and adults with or without specific deficits [4,5,6,7,8]. GVT is an indirect psychometric measure of oculomotor performance, used for the clinical assessment of oculomotor behavior.

The original GVT test [3] consists of two cards with five contorted lines of increasing overlap, crowding, and difficulty. The subject starts from each of the letters at the top of the page, follows the line from the letter to the corresponding number at the bottom of the page, and names the number. Despite the fact that the GVT test was originally intended for use on subjects in the developmental age range, it can also be applied to adults [5]. Individuals with visual and cognitive deficits following different etiologies, such as traumatic brain injury (TBI) or acquired brain injury (ABI) [6,9,10], can exhibit oculomotor alterations. It has been used in an adapted form in different studies involving healthy [4,8,11], learning disabled [12], epileptic [13,14,15], and occipital injured [16] children to assess visual perceptual abilities.

Recently, Zee [17] drew the attention of the neurology community involved in examining eye movement disorders in various neurological deficits to the requirements for and availability of easy-to-use tools to be used to measure and quantify such conditions. Oculomotor deficits can be found in patients who have suffered a stroke at a high percentage, ranging from 7% to 86% [18,19,20,21], depending on specific deficits, the time elapsed since the stroke, and the stage of recovery. In particular, oculomotor problems have been observed in association with specific cognitive deficits such as unilateral spatial neglect [22,23], neglect dyslexia [24,25], simultanagnosia [26], oculomotor apraxia [27], Balint syndrome [28], progressive supranuclear palsy (PSP) [29], and cerebellar ataxia [30].

Tests of oculomotor functioning such as GVT, which although may appear to be limited in comparison with the recording of eye movements, are becoming promising tools for use for the fast evaluation of eye movement disorders. They can be used either with neurologically unimpaired individuals or neurological patients, in clinical contexts where eye-tracking technology is not suitable because of the difficulty of implementation [31,32,33]. From a clinical point of view, only a few simple paper-based tasks for oculomotor functioning on are clinically available, and those that have been proposed have some limitations in the normative values that are available [3,32,34,35,36]. The available oculomotor tests differ in their characteristics; therefore, they may not address the same aspects of oculomotor behavior [31].

Only one study has been directed towards the assessment of the psychometric properties of the original GVT test that are necessary for its correct clinical use. That study showed that the original five-line version is useful for adults but too difficult for young children, for which an easier three-line modified version is more appropriate [5]. In any case, for clinical application, GVT lacks reference norms for adults.

Consequently, the aim of this study has been to assess the impact of age on eye tracing behavior and to define specific normative data for the GVT test with the application of a new scoring system.

## 2. Materials and Methods

### 2.1. Subjects

A power analysis was first performed to assess the minimum sample size required. Because the definition of normative values was regression-based, we followed this approach for the power analysis (see statistical methods paragraph for details). Based on a regression model with three independent factors (demographic characteristics: age, education, and sex), alpha of 0.05, power of 0.80, and effect size f^2^ of 0.04, we determined a minimum sample size required of 277 participants.

A group of 537 participants was originally enrolled, but because of the presence of extreme outliers (3 × IQR over the third quartile) in the execution times, 11 participants were removed, giving a final sample of 526 participants. The procedure used for filtering is described in the section dealing with statistical methods. The participants had a mean age of 45.9 years (SD 16.0, range 20–79). The education mean was 13.41 years (SD 3.7, range 5–25). Of 526 participants, 292 were females (56%). These were subdivided into six age groups, increasing in decades, from 20–29 to 70–79 years old. The size of our sample for each decade, compared with the age distribution of the 40–79 years old adult Italian population in 2020, was not significantly different (χ^2^_(5)_ =2.47, *p* = 0.78). Participants were recruited as a convenience sample from those available by direct contact from all examiners. Table 1 summarizes the demographic data of the participants.

The inclusion criteria were the presence of normal binocular vision assessed by the cover test, the absence of ocular diseases reported by the participants, and a visual acuity equal to or greater than +0.1logMAR in each eye, at near, using SLOAN letters logMAR chart (Goodlite 729000, Elgin, IL, USA). The exclusion criteria were the actual or previous presence of neurological or psychiatric disorders reported by the participants.

Before the evaluation, the participants signed informed consent in order to participate in the study. The study was carried out following the guidelines given in the Declaration of Helsinki, and it was approved by the Optics and Optometry Institutional Review Board of the University of Milano-Bicocca (5/2019; 13 May 2019).

### 2.2. Groffman Visual Tracing Test

**Figure 1:**
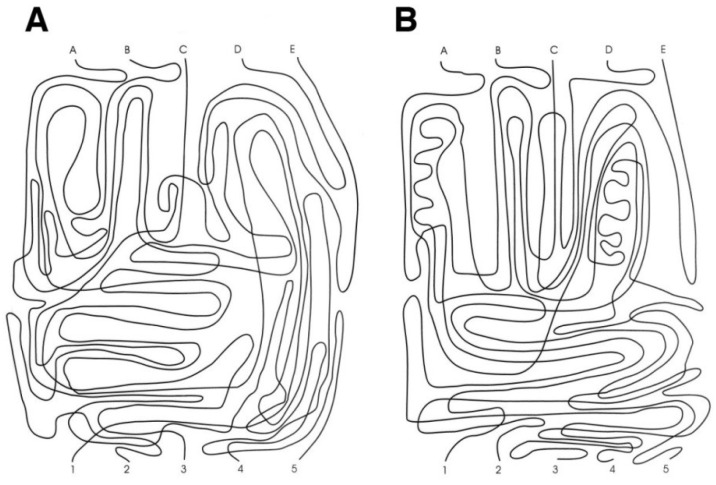
Groffman visual tracing test. The demonstration card was not shown. Panels (**A**) and (**B**) show respectively the card A and B.

Following the original instructions [3], the GVT test is composed of two cards of 216 × 279 mm (i.e., US letter size, Figure 1). Each card consists of five separate intersected continuous lines in a twisted pattern. The task consists of rapidly and accurately “following with the eyes” each line without losing it. The task requires starting from each of the letters at the top of the page (A, B, C, D, and E), following the line from the letter to the corresponding number at the bottom of the page (1 to 5), and naming the number. The corresponding number and the execution times are both recorded. As a pre-test, the demonstration card is shown to the participant, and the instructions about the start, intersections, and ends are explained carefully. The demonstration card is intended to enable the instructions to be understood and to check that the subject possesses the minimum skills required to execute the test. When a participant could not follow a single line on the demonstration card correctly after three attempts, testing was halted because the required level of the minimum skill had not been attained.

As reported in the original paper, the instructions were: *“This is a test to see how quickly and accurately you can follow a line using only your eyes. Look at the line that starts at the letter A, Follow it with your eyes. When it reaches another line (point to the first intersection), follow it through the gap (point to the broken line). This line goes under the whole line and continues through.”* (Groffman, 1966, p. 140). After the demonstration card, cards A and B were always administered in the same order. The instructions for each card and line were: *“Now we are going to trace five more lines. Your score will depend on accuracy and speed, so work quickly, but try not to make a mistake.”* (Groffman, 1966, p. 140). The answer keys for cards A and B were reported on the scoresheet.

### 2.3. Procedure

The evaluation was performed in a quiet and well-illuminated room (about 350–400 lux). Initially, consent to participate in the research was signed by participants, and the inclusion/exclusion criteria were checked. Each participant was seated at a desk wearing the correct glasses (if necessary), and the different cards were positioned on a lectern at a distance of 40 cm. A stopwatch was used to record the execution time. The first card A was positioned on the lectern, and the lines were covered by a white sheet to prevent the participant from following the lines before starting the test. Consequently, only the five letters at the top of the page were visible. The examiner named the first letter removed the white sheet and started recording the time. When the participant named the corresponding number, the examiner stopped the stopwatch. The accuracy (i.e., number of lines followed correctly) and the execution times were recorded on the scoresheet. For each line, if the number reported was not correct, accuracy was scored as zero, and only if the number reported was correct was the execution time recorded, and the accuracy for the tested line was 1. If the participant lost the mark, the accuracy was zero. Scoring of the GVT test was performed using the overall accuracy and mean execution time of each card and line (2 cards × 5 lines) [5].

### 2.4. Statistical Methods

When plotting the raw data of execution times, some high outliers emerge for one line. It is possible that the participant could have gone back or restarted the task, and the examiner could not have recognized this behavior, even if it was not admitted. For this reason, a posteriori case-wise deletion of univariate extreme outliers was performed. Based on all execution times, the non-parametric threshold for the extreme outlier was calculated as three times the interquartile range (3 × IQR) over the 3rd quartile [37]. The value obtained was 78 s. If the execution times of at least one line were equal to or greater than 78 s, all data for the individual participant were discarded. This corresponds to a case-wise deletion of 11 participants, from 537 to 526.

Initially, a series of descriptive and inferential analyses were performed to evaluate the performance of the GVT test over age groups with respect to accuracy and execution times. Comparisons of accuracy between age groups were performed with 1-way ANOVA. Accuracy was measured using a score from 0 to 10. Since not all participants performed all lines correctly, the comparisons of execution times were performed with a linear mixed model (LMM) ANOVA using Id (anonymous identities) as a random factor (random intercept) and Card, Line, and Group as fixed factors with all interactions.

The definition of normative values was performed using a standard procedure used in neuropsychological testing [38,39,40]. To judge whether a participant performs at a normal level in a specific test, it is necessary to compare its performance to the population sample with the same demographic characteristics. This procedure requires collecting data for each factor that influences the score. Consequently, a very large sample, with a minimum of 90–100 participants for each category of gender, age, and education level is needed, resulting in thousands of participants. An efficient alternative model is to subtract the influence of age, gender, and education (if necessary [41]) from the raw score and to calculate the normative data on this adjusted score using a non-parametrical approach [39]. This scoring system was widely used in the field of neuropsychological testing and requires only some hundreds of participants [42,43,44,45,46].

Based on the results of the previous analyses, irrespective of whether the comparisons between lines and cards were significant, execution times were scored whether they were separated or not. The final goal was to make the differences between lines uniform and to have the same mean execution time for all lines. The influence of the line on the execution time was balanced using the steps outlined below. Firstly, the mean execution time of each line for all participants was calculated. Secondly, the mean value of these means was calculated. The difference between the mean of each line from the mean of the means was determined. These series of values (one for each line), with reversed signs, represented the first correction factor and were added to the raw data for the execution time of each participant. A table that could be used to facilitate calculation was provided. Thirdly, since the participants may have followed a different number of lines (from 1 to 10) correctly, a mean execution time for each participant was calculated. This scoring procedure provided two easy scores for GVT, namely accuracy and execution time.

Following this procedure, the influence of demographic variables (age, education, and gender) on the dependent variable (mean corrected execution times or accuracy) was assessed in different steps.

1. Using the general linear model, a series of bivariate regressions were performed, with different transformations of the independent variable (age, education, sex) to find the most appropriate transformation [38,39]. The transformations used were: linear, reverse, quadratic, logarithmic, logarithmic reverse, square root, geometrical, inverse, and exponential.
2. Akaike’s Information Criterion (AIC) [47] was used for the selection of the most appropriate transformation model for each independent variable [48].
3. The three best bivariate models (one for each predictor) were entered into a multivariate model with two or three independent factors.
4. We used AIC model selection to find the most appropriate model among a set of 7 possible models describing the relationship between the dependent variable (accuracy or execution time) and age, education, and sex in their single or multiple combinations.
5. Subsequently, based on the previous result, a second regression model was built, based on deviation from the mean. Then, by reversing the regression coefficients, a regression for adjusting the score was calculated taking into account the contribution of each confounding variable. The two regressions discussed above are not equivalent because the first one used the raw score as a dependent variable. In contrast, the second one used the deviation from the mean. For its clinical usefulness, only the second model was reported.
6. Based on the results of this regression, a simple correction grid was built to facilitate the scoring process. Specifically, since from a clinical point of view it is easier to find age and education in a table when the value falls in a specific range (e.g., 20–29), the age included in the regression was the mean of the interval considered (e.g., 24.5). This represents a simplification, but the correction grid is a simpler tool to facilitate clinical use. A precise detailed scoring could be performed using the regression equations.

In order to define a cut-off score, the one-sided non-parametric 95% tolerance intervals, with a confidence limit of 95%, were then calculated. For accuracy, the leftward limit was calculated and for the execution time, the rightward limit was considered. Corrected scores, percentile, and rank-based equivalent scores [49] were calculated and reported for clinical use. Statistical analyses and figures were performed with R statistical environment 4.0.3 [50] and specific packages: ez 4.4-0 [51], Hmisc 4.6-0 [52], lme4 1.1-26 [53], lmerTest 3.1-3 [54], Tolerance 2.0.0 [55], and AICcmodavg 2.3.1 [56].

## 3. Results

### 3.1. Descriptive Analyses of GVT

#### 3.1.1. Accuracy

**Figure 2:**
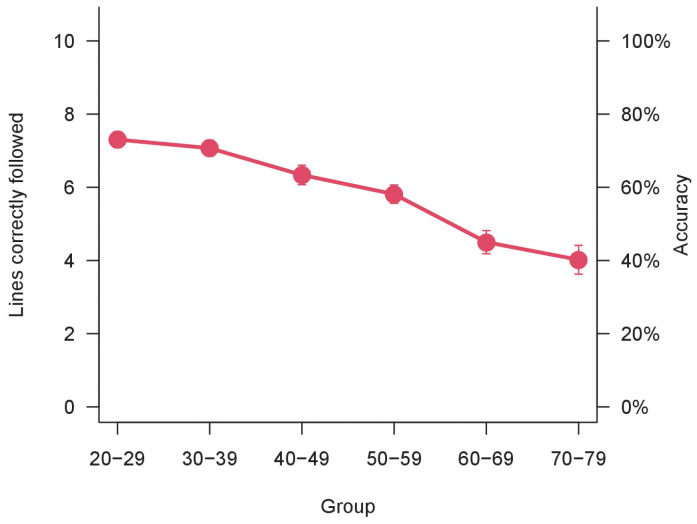
Accuracy over age groups. Bars represent ±1 SEM.

The descriptive data of the sample acquired are reported in Table 2. The results of the one-way ANOVA on accuracy were significant (F_(5,520)_ = 21.54, *p* < 0.001, η^2^_p_ = 0.17). This result was confirmed by the non-parametrical Kruskal–Wallis rank-sum test (χ^2^_(5)_ = 83.25, *p* < 0.0001). The accuracy decreases over age groups, as illustrated in Figure 2.

**Figure 3:**
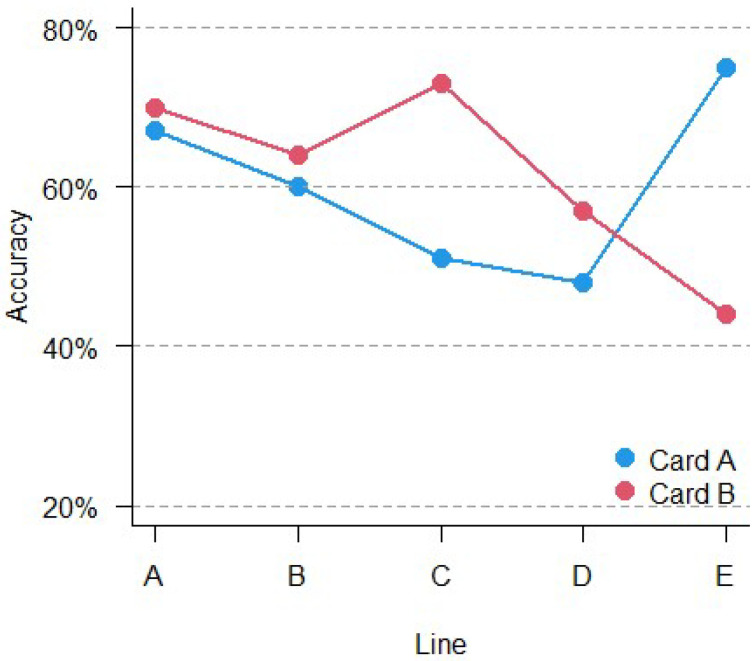
Accuracy comparison over card and lines.

In the second line of Table 3, the number of the lines that were not followed correctly in the total of 526 participants are reported as invalid. The data listed in Table 3 show that different lines have a different level of accuracy. To assess the different levels of accuracy between lines, the comparison was performed using the χ^2^ test, which revealed significant differences in accuracy between lines. Each line presents different levels of difficulty (χ^2^_(9)_ = 88.87, *p* < 0.001). The results are shown in Figure 3. However, since only the overall accuracy was considered for clinical purposes, this result was reported only for exhaustiveness.

#### 3.1.2. Execution Times

The descriptive statistics of the different execution times separated for cards and lines are listed in Table 3.

**Figure 4:**
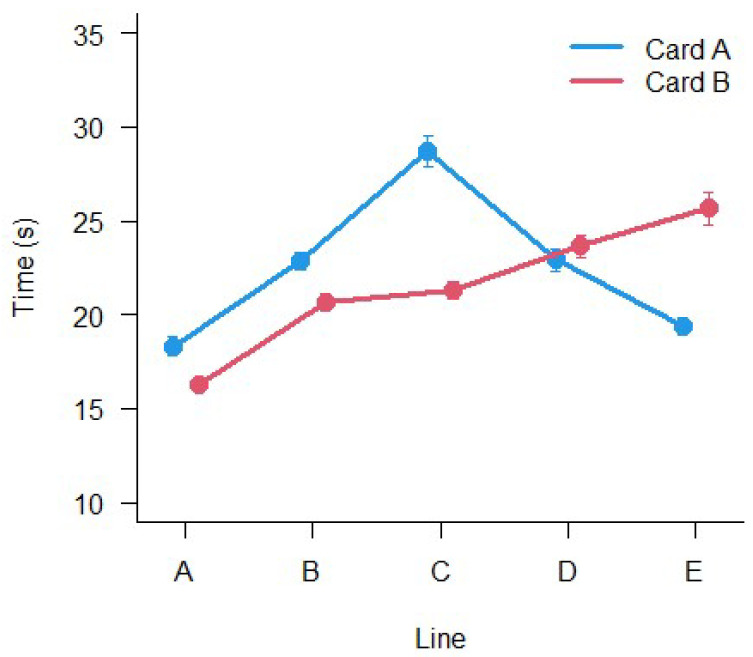
Mean execution times between the different lines (A–E) and the two Cards. Bars represent ±1 SEM.

**Figure 5:**
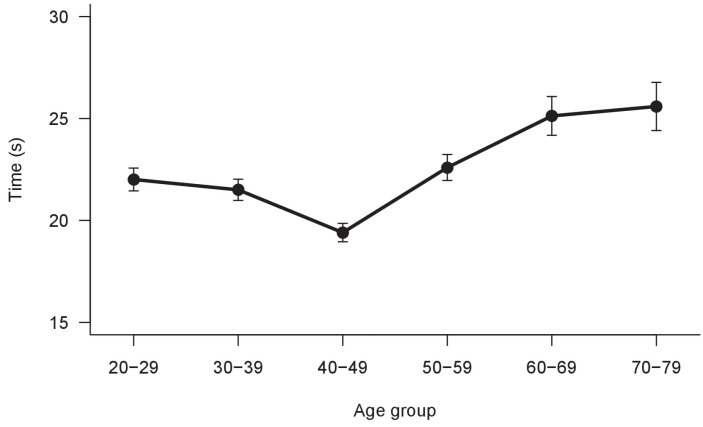
Mean execution times between age groups. Bars represent ±1 SEM.

The results of LMM ANOVA show a significant main effect of Card (F_(1,2761)_ = 7.89, *p* < 0.005), a significant main effect of Line (F_(4,2770.4)_ = 79.67, *p* < 0.001), a significant main effect of Group (F_(5,485.9)_ = 8.27, *p* < 0.001) and a significant interaction Card × Line (F_(4,2759)_ = 53.12, *p* < 0.001). No other interactions were significant. The results are shown in Figure 4 and Figure 5. The execution times are different between all lines and cards and, overall, they change with age.

### 3.2. Definition of Normative Data

#### 3.2.1. Accuracy

Firstly, different bivariate regressions were tested to assess the influence of demographic variables. A series of bivariate regressions were performed to find the most appropriate transformation of independent variables (see statistical method paragraph for details). The models with lower AIC were included in the comparison between bivariate and multivariate models. The results of comparisons of the bivariate and multivariate models are shown in Table 4.

The results showed that the best model, carrying 78% of the cumulative model weight, included age, education and gender. The regression for correction of accuracy (AC) consequently is:

AC = 0.000597 × (Age^2^ − 2365.458) + 16.77 × (1/Education − 0.0818) − 0.455 × (Sex − 0.445) (1)

where sex F = 0 and M = 1. The model has an adj R^2^ of 0.194. For an easy clinical application, a correction grid was built from regression (1), and it is given in Table 5.

Decimal values were added to obtain precise scoring on corrected values. Subsequently, on the corrected score, the lower 95% one-side tolerance intervals with 95% confidence intervals were calculated. The results indicated 1.6 for the outer limit and 2.1 for the inner limit. The scores between these two values represent uncertainty. Rank-based equivalent scores (ES) and percentile scores were calculated, and they are reported in Table 6 and Table 7, respectively.

#### 3.2.2. Execution Times

Since execution times were influenced by Age, Line, and Card, and the scoring line by line was time-consuming with the difficulty of interpretation, a slightly different approach was used. It was based on different steps. Firstly, a correction grid was constructed to make the execution times across lines uniform. This was performed simply by changing the sign of the difference from the mean time of execution of each line (Figure 4) from the mean of the means of execution times. The results are listed in Table 8.

Secondly, since the participants could follow correctly more than one line, the mean (corrected) execution time for each one was calculated. The mean corrected times were then checked to find the most appropriate transformation of the demographic variables. This was done using the same procedure described for accuracy and detailed in the statistical method section.

After that, the most effective bivariate models were compared to their combination in multivariate models. Results are shown in Table 9.

The results of regression comparison showed that the best model, carrying 43% of the cumulative model weight, included only age. The regression for correction of execution time (TC) is:

TC = 4.364 × (log (86.9 − Age) − 3.7)(2)

with an R^2^ of 0.07. To obtain a straightforward clinical application, a simple correction grid from (2) was built. This is given in Table 10.

The calculation of the upper 95% one-side tolerance intervals, with 95% confidence intervals on the two steps corrected scores, provided a result of 34 s for the inner and 37.4 s for the outer limit. The scores between these two values represent the uncertainty. Rank-based equivalent scores and percentiles were calculated and are listed in Table 11 and Table 12, respectively.

### 3.3. Examples to Illustrate the Scoring of GVT

For illustration purpose, two examples were outlined. The first consider a case where a 48-year-old woman with a university education finishes the GVT with the scores shown in Table 13 and Table 14. She correctly followed all lines.

She showed perfect accuracy and a median execution time. The subject performed the task the most accurately, achieving a median result in terms of speed. In general, this is normal behavior.

The second example shown in Table 15 and Table 16 is from a man of 23 years old with 13 years of education.

Only three lines were followed correctly. As is evident in the last two columns of Table 16, the accuracy percentile score was very low, bordering on a pathological score. Nevertheless, the speed of execution was extremely high, performing well over the mean. This case could represent a subject who performs faster but with low accuracy.

## 4. Discussion

The aim of this study was to assess the influence of age on visual tracing performance by using the GVT task and to provide adult norms for this test. Scoring based on the overall accuracy and execution times has been applied as a standard in many neuropsychological performance tests [31,43,57,58].

The results show that accuracy decreases over age groups. This represents a clear aging trend. Each line on a different card showed a specific accuracy level which was slightly but significantly different one to the others. However, this is an intrinsic characteristic of the test, and there are no floor or ceiling effects that invalidate the task.

Execution times, other than increasing with age, as previously shown in a small number of participants [5], have been shown to be different for each line and card. The previous result has been confirmed in the current study with a larger and more representative sample, which was necessary for defining norms.

There is an awareness that there are many cognitive factors that influence the performance of the oculomotor test, primarily visuospatial attention [1,2,4,59]. Nonetheless, paper-based oculomotor tests could be helpful in many clinical situations [18,31,33].

Normative data were produced, keeping in mind the procedure usually used in the neuropsychological tests. Accuracy was influenced by age, education, and sex, while mean execution time was influenced only by age. With a specific adaptation for obtaining mean execution time, the results are reported as percentiles and equivalent scores for different clinical requirements. Even though this process of scoring seems time-consuming, it represents a standard in neuropsychological testing and allows a comparison to be made of the scores obtained with other tests that use the same standard scores, namely percentile or equivalent score.

Although the test includes two cards and five separated lines, it is advantageous to consider it as a whole, in particular with respect to accuracy. This takes into account that the accuracy over 10 lines represents a better scoring method than considering separate scoring for each line and card (5 + 5). Conversely, for execution times, a slightly complex method of scoring has been applied because of the nature of the task itself (execution time is available only for the lines followed correctly) and to obtain a single (mean) score of execution times. Alternatively, each line needs to be scored separately, giving a series of speed scores, one for each line followed correctly. This procedure in a clinical setting is time-consuming, as well as making it difficult to interpret multiple results. By using the method of scoring applied in this study, a simple assessment of speed and accuracy can be performed.

This study has set the basis for clinical application of the GVT test in the adult population. Future directions could involve its use on specific populations of neuropsychological patients such as ABI and TBI, and the comparison of GVT with either eye-tracking or other paper-based oculomotor tests, such as King Devick, the DEM test, and the visual search test [31].

The participants were from Italy, and consequently, the norms could be correctly defined as Italian norms. However, since in this test, as in many visuospatial tasks, there is no influence of culture or language, in the absence of other studies, they can be used as an independent international reference. It is important to note, however, that the norms presented have some limitations (and uncertainty). In another sample of the same size, the model used to calculate adjusted scores and its coefficients may differ depending on the specific sample. In future normative studies, a representative and larger sample could be used to verify and ameliorate this point.

## 5. Conclusions

The ability to follow a line with the eyes is influenced by age. The GVT test is a simple tool for the assessment of eye movement behavior and now, with reference values, it can be used in a clinical setting in healthy-adult and neurological populations.

## Figures and Tables

**Table 1:** Demographic characteristics of the participants’ sample. F = female; M = male.

Age	20–29		30–39		40–49		50–59		60–69		70–79		Tot.
School	F	M	F	M	F	M	F	M	F	M	F	M	
0–5	0	0	0	0	0	1	0	0	0	1	4	2	8
6–8	0	0	3	6	5	5	11	10	11	6	9	12	78
9–13	16	12	18	20	21	16	26	19	21	15	6	9	199
>13	47	27	33	21	29	24	21	14	8	8	3	6	241
Tot.	63	39	54	47	55	46	58	43	40	30	22	29	526

**Table 2:** Descriptive results of accuracy on the GVT test separated for decades of age.

Age Group	20–29	30–39	40–49	50–59	60–69	70–79
Mean	7.3	7.07	6.34	5.81	4.5	4.02
SD	2.17	2.2	2.67	2.5	2.64	2.8
Median	8	8	7	5	4	3
Max	10	10	10	10	10	9
Min	2	1	0	0	0	0

**Table 3:** Descriptive statistics of execution times for the GVT test separated for each Card and Line. Data are reported in seconds. n. Valid = number of the lines followed correctly; n. Invalid = lines followed incorrectly, missed or abandoned by the participants; Total = total number of participants.

Card	A					B				
Line	A	B	C	D	E	A	B	C	D	E
n. Valid	354	317	268	255	392	369	336	386	302	231
n. Invalid	172	209	258	271	134	157	190	140	224	295
Mean	18.3	22.9	28.7	22.9	19.4	16.4	20.7	21.3	23.6	25.7
Std.dev	8.9	8.6	13	9	9.4	6.8	8.4	8.9	9.9	13
Median	15.9	21	25	20,8	17	14.5	19	19	21	22
Min	5	6	9.4	8.9	6.9	5	7.6	8	9	5.7
Max	71	66	76.7	60.1	70.3	58	62.8	70	77	74.7
Total	526	526	526	526	526	526	526	526	526	526

**Table 4:** Comparison between regression models with the best transformation of independent variables for accuracy. K = Number of parameters of the model; AICc = Akaike’s Information Criterion corrected; Delta AIC = AIC difference between the best model and the model listed; Model Lik. = the relative likelihood of the model; AICc Wt = model probabilities; LL = log-likelihood of the model; Cum. Wt = cumulative Akaike weights.

Model	K	AICc	Delta AICc	Model Lik.	AICc Wt	LL	Cum. Wt
Age + Edu + Sex	5	2429.72	0	1	0.78	−1209.8	0.78
Age + Edu	4	2432.25	2.53	0.28	0.22	−1212.09	0.99
Age + Sex	4	2443.36	13.64	0.001	<0.001	−1217.64	1
Age	3	2444.99	15.26	<0.001	<0.001	−1219.47	1
Edu + Sex	4	2485.29	55.56	<0.0001	<0.0001	−1238.61	1
Edu	3	2486.68	56.96	<0.0001	<0.0001	−1240.32	1
Sex	3	2541.07	111.35	<0.0001	<0.0001	−1267.52	1

**Table 5:** Correction grid for accuracy on GVT.

Sex	Female				Male			
Education	0–5	6–8	9–13	>13	0–5	6–8	9–13	>13
20–29	4.5	0.2	−0.7	−1.2	4.0	−0.3	−1.2	−1.6
30–39	4.8	0.5	−0.3	−0.8	4.4	0.1	−0.8	−1.3
40–49	5.3	1.0	0.1	−0.4	4.9	0.5	−0.3	−0.8
50–59	5.9	1.6	0.7	0.2	5.4	1.1	0.3	−0.2
60–69	6.6	2.3	1.4	0.9	6.2	1.8	1.0	0.5
70–79	7.4	3.1	2.3	1.8	7.0	2.7	1.8	1.3

**Table 6:** Equivalent scores for corrected values of GVT accuracy.

ES	Corrected Score
0	≤1.6
1	1.7–3.7
2	3.8–5.1
3	5.1–6.3
4	>6.3

**Table 7:** Percentiles for corrected scores of GVT accuracy.

Percentile	Corrected Score
99	10.9
95	9.7
90	9.1
85	8.7
80	8.3
75	8.0
70	7.6
65	7.3
60	6.9
55	6.6
50	6.3
45	5.9
40	5.5
35	5.2
30	4.8
25	4.4
20	3.9
15	3.4
10	2.7
5	2.0
4	1.8
3	1.6
2	1.0
1	0.7

**Table 8:** Correction grid aimed to uniform the mean performance on the execution times for each line and card.

CARD	A					B				
LINE	A	B	C	D	E	A	B	C	D	E
Correction Value	+3.6	−0.9	−6.7	−0.9	+2.6	+5.7	+1.3	+0.7	−1.7	−3.7

**Table 9:** Comparison between regression models with the best transformation of independent variables for the mean execution time. K = Number of parameters of the model; AICc = Akaike’s Information Criterion corrected; Delta AIC = AIC difference between the best model and the model listed; Model Lik. = the relative likelihood of the model; AICc Wt = model probabilities; LL = log-likelihood of the model; Cum. Wt = cumulative Akaike weights.

Model	K	AICc	Delta AICc	Model Lik.	AICc Wt	LL	Cum. Wt
Age	3	3451.33	0	1	0.43	−1722.64	0.43
Age + Sex	4	3452.32	0.99	0.61	0.26	−1722.12	0.68
Age + Edu	4	3452.9	1.58	0.45	0.19	−1722.41	0.88
Age + Edu + Sex	5	3453.82	2.50	0.29	0.12	−1721.85	1
Edu	3	3479.66	28.33	<0.0001	<0.0001	−1736.81	1
Edu + Sex	4	3480.92	29.59	<0.0001	<0.0001	−1736.42	1
Sex	3	3490.42	39.09	<0.0001	<0.0001	−1742.19	1

**Table 10:** Correction grid for age on execution times.

Age Range	Correction Value
20–29	+1.9
30–39	+1.1
40–49	+0.2
50–59	−1.0
60–69	−2.6
70–79	−5.2

**Table 11:** Equivalent scores for corrected execution times.

ES	Corrected Execution Time
0	≥37.4
1	37.3–28.1
2	28.8–23.9
3	23.8–20.8
4	≤20.7

**Table 12:** Percentiles for corrected execution times.

Percentile	Corrected Execution Time
99	10.3
95	13.4
90	14.7
85	15.8
80	16.6
75	17.2
70	17.8
65	18.5
60	19.2
55	20.1
50	20.7
45	21.5
40	22.8
35	23.8
30	25.0
25	26.1
20	27.8
15	29.7
10	32.0
5	35.0
4	36.5
3	37.7
2	40.4
1	42.8

**Table 13:** Example 1 of application of GVT in one case: raw scores.

Card	Card A					Card B				
Line	A	B	C	D	E	A	B	C	D	E
Raw score	19.4	21.0	33.9	23.9	18.4	16.5	13.3	19.7	20.3	19.7
1st correction	*+3.6*	*−0.9*	*−6.7*	*−0.9*	*+2.6*	*+5.7*	*+1.3*	*+0.7*	*−1.7*	*+3.7*
Adj. score	23	20.1	27.2	23	21	22.2	14.6	20.4	18.6	23.4

**Table 14:** Example 1 of application of GVT in one case: scoring.

	Value	Demographic Correction	Corrected Value	Percentile	ES
Accuracy	10	−0.4	9.6	−95	4
Execution times	21.4	+0.2	21.6	−45	3

**Table 15:** Example 2 of application of GVT in one case: raw scores.

Card	Card A					Card B				
Line	A	B	C	D	E	A	B	C	D	E
Raw score	12.8					12.7			14.4	
1st correction	*+3.6*	*−0.9*	*−6.7*	*−0.9*	*+2.6*	*+5.7*	*+1.3*	*+0.7*	*−1.7*	*+3.7*
Adj. score	16.4					18.4			12.7	

**Table 16:** Example 2 of application of GVT in one case: scoring.

	Value	Demographic Correction	Corrected Value	Percentile	ES
Accuracy	3	−1.2	1.8	4	1
Execution times	15.8	+1.9	17.7	70–75	4

## Data Availability

The data presented in this study are available on request from the corresponding author. The data are not publicly available due to restrictions included in the informed consent provided by participants.
